# Development of risk prediction nomogram for neonatal sepsis in Group B Streptococcus-colonized mothers: a retrospective study

**DOI:** 10.1038/s41598-024-55783-2

**Published:** 2024-03-07

**Authors:** Mengqi Wu, Yanbing Deng, Xinye Wang, Baomei He, Fangqiang Wei, Ying Zhang

**Affiliations:** 1 Center for Reproductive Medicine, Department of Pediatrics, Zhejiang Provincial People’s Hospital, Affiliated People’s Hospital, Hangzhou Medical College, Hangzhou, 310014 Zhejiang China; 2 Department of Postgraduate Education, Jinzhou Medical University, Jinzhou, 121004 Liaoning China; 3 General Surgery, Cancer Center, Department of Hepatobiliary & Pancreatic Surgery and Minimally Invasive Surgery, Zhejiang Provincial People’s Hospital, Affiliated People’s Hospital, Hangzhou Medical College, Hangzhou, 310014 Zhejiang China

**Keywords:** Group B Streptococcus, Intrapartum antibiotic prophylaxis (IAP), Neonatal infection, Risk factors, Nomogram prediction model, Risk factors, Paediatrics, Medical research, Paediatric research, Bacterial infection, Neonatal sepsis

## Abstract

Neonatal clinical sepsis is recognized as a significant health problem, This study sought to identify a predictive model of risk factors for clinical neonatal sepsis. A retrospective study was conducted from 1 October 2018 to 31 March 2023 in a large tertiary hospital in China. Neonates were divided into patients and controls based on the occurrence of neonatal sepsis. A multivariable model was used to determine risk factors and construct models.The utilization and assessment of model presentation were conducted using Norman charts and web calculators, with a focus on model differentiation, calibration, and clinical applicability (DCA). Furthermore, the hospital’s data from 1 April 2023 to 1 January 2024 was utilized for internal validation. In the modelling dataset, a total of 339 pairs of mothers and their newborns were included in the study and divided into two groups: patients (n = 84, 24.78%) and controls (n = 255, 75.22%). Logistic regression analysis was performed to examine the relationship between various factors and outcome. The results showed that maternal age < 26 years (odds ratio [OR] = 2.16, 95% confidence interval [CI] 1.06–4.42, p = 0.034), maternal gestational diabetes (OR = 2.17, 95% CI 1.11–4.27, p = 0.024), forceps assisted delivery (OR = 3.76, 95% CI 1.72–5.21, p = 0.032), umbilical cord winding (OR = 1.75, 95% CI 1.32–2.67, p = 0.041) and male neonatal sex (OR = 1.59, 95% CI 1.00–2.62, p = 0.050) were identified as independent factors influencing the outcome of neonatal clinical sepsis. A main effects model was developed incorporating these five significant factors, resulting in an area under the curve (AUC) value of 0.713 (95% CI 0.635–0.773) for predicting the occurrence of neonatal clinical sepsis. In the internal validation cohort, the AUC value of the model was 0.711, with a 95% CI of 0.592–0.808. A main effects model incorporating the five significant factors was constructed to help healthcare professionals make informed decisions and improve clinical outcomes.

## Introduction

Group B Streptococcus (GBS), also known as Streptococcus agalactiae, is a Gram-positive bacterium that commonly colonizes the human reproductive and gastrointestinal tracts^1^. GBS colonization in the maternal genital tract (vagina and perianal area) can cause neonatal sepsis through intrauterine infection or vaginal delivery^2^. Neonatal sepsis caused by GBS is a common infectious disease in newborns, with the most common manifestations being bacteremia, sepsis, pneumonia, and/or meningitis without an identifiable focus of infection^3^. Adverse outcomes include long-term disabilities (blindness, deafness, and cerebral palsy) and death. However, clinical neonatologists face difficulties identifying the course of GBS neonatal sepsis and determining the optimal timing for administering antibiotics. In several European and American countries, sensitive antibiotics are administered to newborns immediately after delivery to prevent GBS sepsis. However, the prophylactic use of broad-spectrum antibiotics during delivery may increase the risk of severe late-onset bacterial and antibiotic-resistant infections^4^. Widespread administration of antibiotics to newborns in countries such as China, which have large populations, is inappropriate. In recent years, research on GBS-related vaccines has gained significant momentum^5^. The difficulty in early identification of neonatal GBS sepsis is due to the inconsistent timing of hematological tests such as WBC, CRP, PCT, etc., and the unclear relationship between high and low indicators and the degree of disease progression, making them of limited value for predicting neonatal sepsis^6^. The gold standard for diagnosing neonatal sepsis is based on blood and cerebrospinal fluid cultures, but these methods are time-consuming and have a low positivity rate^7^. It has been reported that the positivity rate of blood culture in suspected early-onset sepsis is only 2–3%^8^, Low sensitivity of gold standard body fluid culture (blood culture, cerebrospinal fluid culture) tests^9^. Therefore, during the initial research design of this study, newborns with clinical manifestations of sepsis and abnormal laboratory data in blood samples after birth were selected as early predictors of sepsis, which not only improved the accuracy of the prediction model but also had greater clinical value.

The predominant emphasis of contemporary neonatal sepsis calculators lies in the examination of variables encompassing antibiotic administration, neonatal ward management, and specific threshold measurements for diverse laboratory examinations^10–12^, alongside the clinical presentation of the infant and the relative risk within the population. Research on neonatal sepsis has shown that perinatal factors in GBS-colonized mothers may be important causes of neonatal sepsis^13^. Herein, we sought to establish a predictive model based on the risk factors for clinical neonatal sepsis caused by factors in GBS-colonized mothers based on data from an integrated obstetrics and neonatology department in a tertiary hospital. We identified the risk factors for clinical neonatal sepsis and measured their impact by assessing a series of maternal, perinatal, and neonatal factors. The identified factors were further used to establish a predictive nomogram for early prediction of neonatal sepsis and to provide recommendations for the clinical use of antibiotics.

## Methods

**Figure 1:**
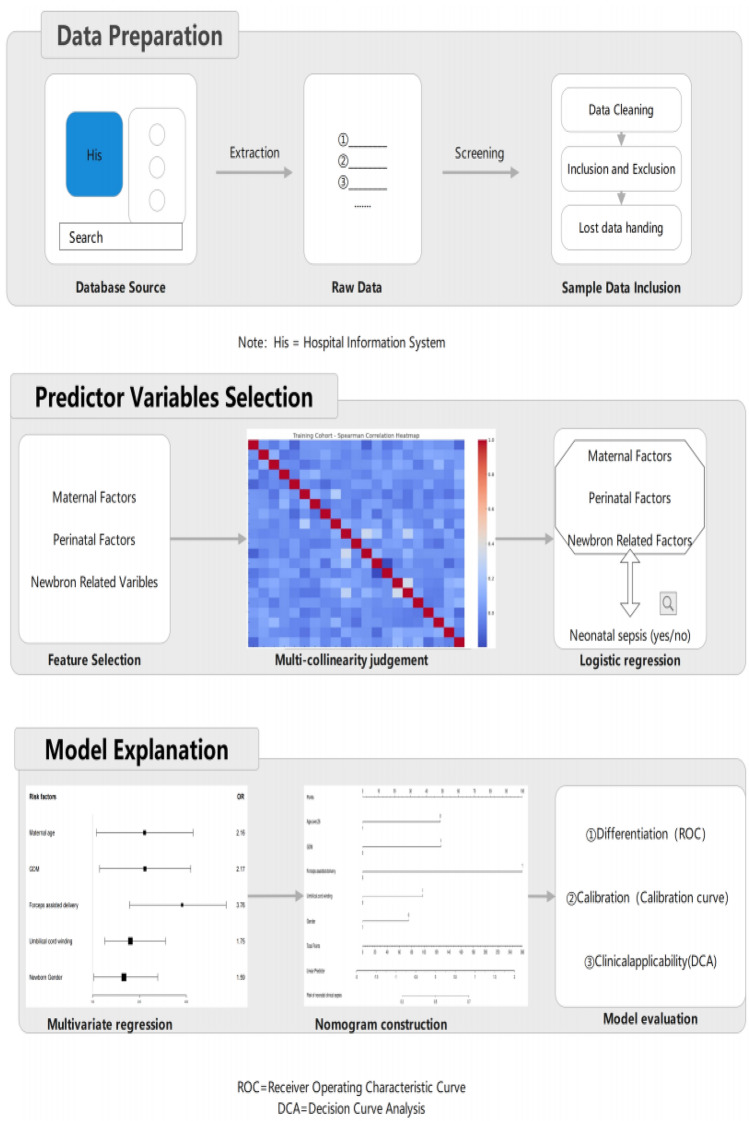
Study workflow diagram.

To provide a comprehensive overview of the study, a detailed workflow diagram was created as a supplementary visual aid, denoted as Fig. 1.

### Data source and study population

**Figure 2:**
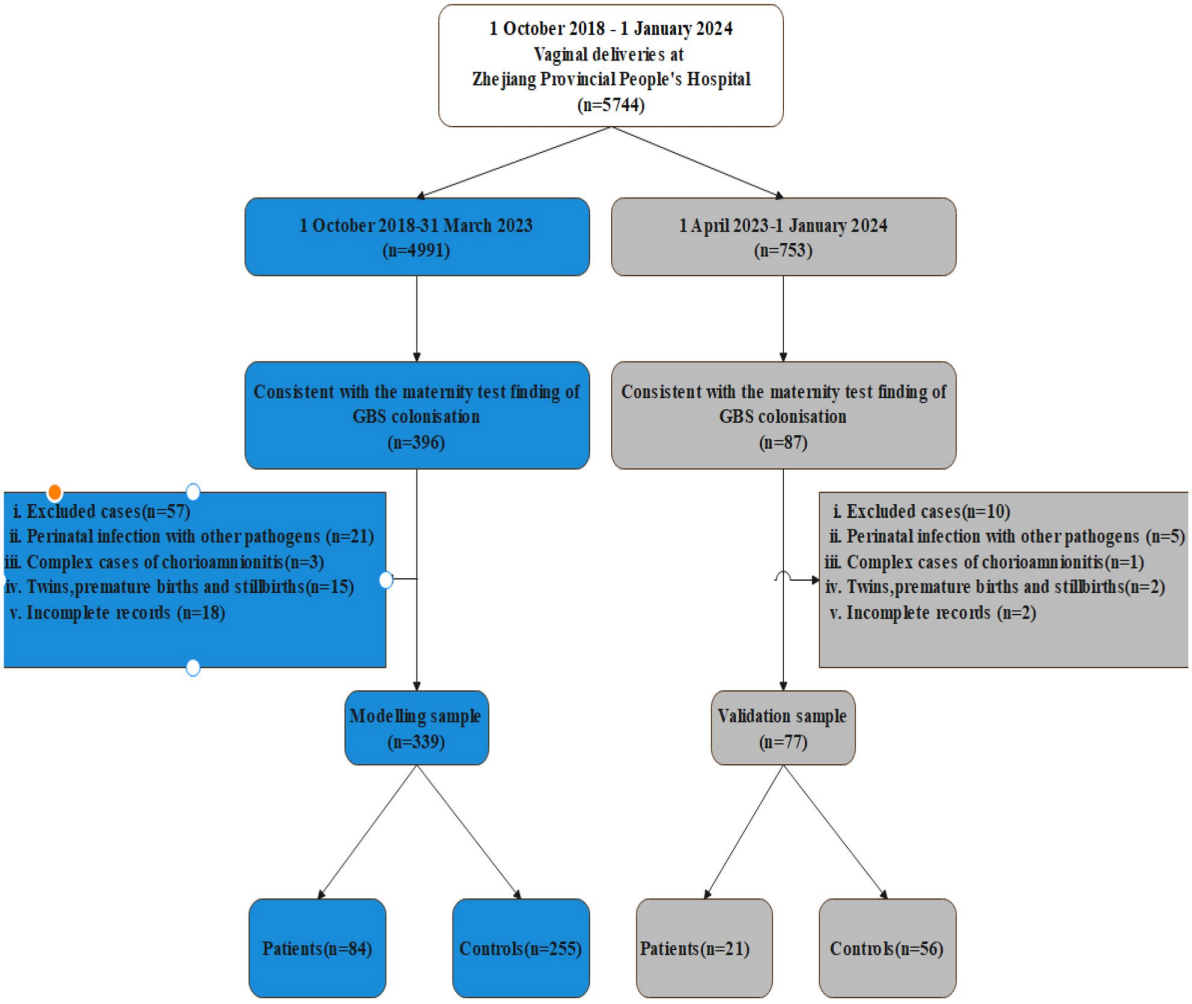
Flow chart for patient selection.

This was a retrospective cohort study, The study cohort was selected from a population of maternal and neonatal mother-baby pairs with GBS colonization, derived from cases of vaginal deliveries at Zhejiang Provincial People's Hospital between 1 October 2018 and 31 March 2023. Infants born prior to 36 weeks of gestation, twin births, and stillbirths were excluded from our study due to their heightened likelihood of requiring invasive medical interventions, which could potentially elevate the risk of early-onset sepsis (EOS) in light of the unique circumstances surrounding their delivery. The exclusion of caesarean section deliveries from our study was based on a thorough examination of data and clinical expertise. This decision was made due to the substantial disparity in the impact of GBS colonisation on neonates between vaginal and caesarean section deliveries. Specifically, GBS colonisation has a more pronounced effect on neonates born via vaginal delivery, and this disparity is both objectively evident and statistically significant.In order to account for the potential influence of concurrent infections in the mother during pregnancy and delivery on the outcome, we opted to exclude instances of chorioamnionitis that were complicated by the presence of other pathogens, such as Chlamydia trachomatis, HIV, and syphilis, during the delivery process. Additionally, we removed 18 cases from our analysis due to incomplete records, ensuring that only samples with complete data were included in our study. It is worth noting that this subset of complete data represented a small proportion of the overall dataset and did not involve the removal of any significant variables. The flowchart depicting the enrollment of the population is presented in Fig. 2.Within the hospital database system, all hospital admissions and obstetric deliveries are tracked through a common medical record number, while laboratory data related to newborns are stored under a subsidiary number linked to the mother's medical number.

#### Description of relevant policies and antibiotic use during labor

Our hospital has a GBS universal screening policy for pregnant women with a documented. The screening is performed during the late stage of pregnancy (between 35 and 37 weeks), and it involves testing for the presence of GBS in the vagina and rectum to confirm the colonization status of the pregnant women. The use of antibiotics during labor follows the guideline, which includes prophylactic antibiotics administered intravenously during labor and as soon as possible after delivery or rupture of membranes. Penicillin is the first-line antibiotic for treating GBS infection in pregnant women. In case of penicillin allergy, alternative antibiotics can be used, including cephalosporins, clindamycin, erythromycin, and vancomycin. Obstetricians and neonatologists provided health education to pregnant women and their families about GBS, ensuring they had a fundamental understanding of the disease and its risks.

Notes 1: (Note 1: The available scientific evidence is limited in terms of quality, and there is a lack of consensus among experts regarding the optimal approach for screening Group B Streptococcus (GBS). However, both risk factor-based screening and routine universal screening are considered acceptable strategies for reducing Early-Onset Group B Streptococcal Disease (EOGBSD). In accordance with our most recent guidelines, it is recommended to implement universal screening between 35 and 37 weeks of gestation, utilizing a sampling method that involves collecting specimens from the lower one-third of the vagina and rectum. The screening validity period should be maintained for a duration of 5 weeks.)

Note 2: The rationale behind selecting data from October 2018 onwards pertains to GBS colonised mothers. The administration of antibiotics during vaginal delivery for GBS colonised mothers in our hospital was appropriately standardized and sufficient after October 2018.

### Variable definition

Based on the literature^14^, the study incorporated a total of 20 risk factors for newborn sepsis, which are presented in Table 1. Indicators of risk are straightforward to assess in clinical settings. In order to construct a nomogram, all the risk factors were transformed into categorical variables; the chosen components comprised general information about the mother, medical history, obstetric records, and neonatal clinical information. The table below categorizes variables into maternal factors, perinatal factors, and neonatal factors. Maternal factors include maternal age, GBS colonisation site, health during pregnancy(HDP/GDM/AIP/HIP), previous history of abdominal surgery, native; perinatal factors including intrapartum fever, intrapartum antibiotic, amniotic fluid, oxytocin induced labor, PROM ≥ 18 h, AROM, forceps assisted delivery, intrauterine distress, umbilical cord winding ;neonatal factors include sex, birth weight, gestational age, birth asphyxia and Apgar score.

**Table 1:** The risk factors with a definition in this study.

Risk factors	Definition
Maternal characteristics	
Maternal age (years)	≥ 26/ < 26
GBS vagina ( +) and perianal ( +)	Yes/no (based on the electronic medical record);
HDP	Yes/no (based on the relevant diagnostic criteria specified in the ACOG guidelines^15–17^)
GDM	Yes/no (based on the relevant diagnostic criteria specified in the ACOG guidelines^15–17^)
AIP	Yes/no (based on the relevant diagnostic criteria specified in the ACOG guidelines^15–17^)
HIP	Yes/no (based on the relevant diagnostic criteria specified in the ACOG guidelines^15–17^)
Previous history of abdominal surgery	Yes/no (including history of surgical procedures on the uterus, ovaries, fallopian tubes and other vital organs of the abdomen)
Native	Yes/no (based on the electronic medical record)
Perinatal characteristics	
Intrapartum fever (℃)	Normal (< 38)
	Abnormal (≥ 38)
Intrapartum antibiotic (times)	< 4
	≥ 4
Amniotic fluid	Normal (Clear; Grade1: lightly stained amniotic; Grade2: green-or-yellow-stained amniotic fluid with some particulate matter)/Abnormal (Grade3: dense meconium with "pea-soup"consistency) (based on the turbidity of the amniotic fluid after the labour process)
Oxytocin induced labor	Yes/no (based on the obstetric documentation)
PROM ≥ 18 h	Yes/no (based on the obstetric documentation)
AROM	Yes/no (based on the obstetric documentation)
Forceps assisted delivery	Yes/no (based on the obstetric documentation)
Intrauterine distress	Yes/no (based on the obstetric documentation)
Umbilical cord winding	Yes/no (based on the obstetric documentation)
Infant characteristics	
Gender	Female/male
Birth weight (g)	2500–4000/ < 2500 and > 4000
GA (weeks)	< 40/ ≥ 40

*GA* gestational age, *AROM* artificial rupture of membranes, *PROM* premature rupture of membrane, *GDM* gestational diabetes mellitus, *HDP* hypertensive disorders of pregnancy, *AIP* anaemia in pregnancy, *HIP* hypothyroidism in pregnancy. GBS vagina( +) and perianal( +) = GBS colonization in the maternal genital tract (vagina and perianal area), Umbilical cord winding was assessed as cord around the neck, cord around the body.

### Variable covariance handling

**Figure 3:**
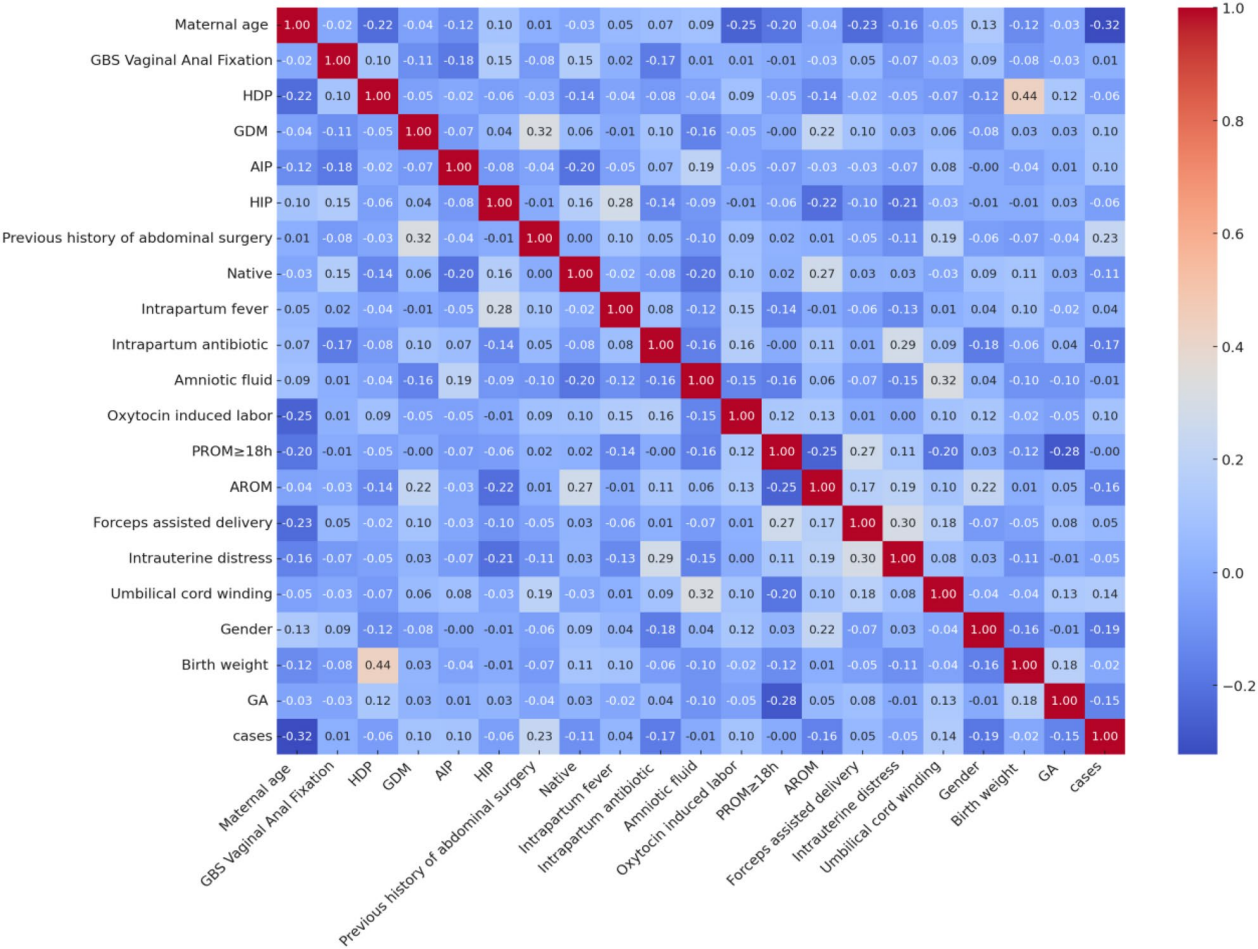
Variable correlation coefficient matrix heatmap.

The variables were subjected to a multicollinearity analysis, wherein the correlation coefficients between each pair of variables were plotted and a heat map of the correlation coefficient matrix was generated. The Fig. 3 depicted that none of the Spearman correlation coefficients between the variables exceeded 0.8, implying the absence of strong correlation among the independent variables. Consequently, the coefficient estimation of the regression model was deemed stable and the hypothesis testing reliable, thereby warranting the inclusion of all variables in the subsequent feature selection stage.

### Outcome—neonatal septicaemia (yes/no)

Integrated Management of Newborn & Childhood Illnesses (IMNCI) clinical diagnostic criteria for neonatal sepsis^15^.

According to this guideline, neonates are recorded as sepsis when they have two or more of the following clinical features along with ≥ 2 subsequent haematological criteria: The presence of persistent fever (≥ 37.5 °C) or persistent hypothermia (≤ 35.5 °C) for a duration exceeding one hour, tachypnoea (≥ 60 respirations per minute), severe chest pumping, gurgling, poor feeding, movement solely in response to stimulation, < $ door rumbling, convulsions, in conjunction with drowsiness or unconsciousness, and the fulfillment of at least two haematological criteria such as total white blood cell count (< 4000 or > 12,000 cells/mm^3^), absolute neutrophil count (< 1500 cells/mm^3^ or > 7500 cells/mm^3^), platelet count (< 150 or > 450 cells/mm^3^), and random blood glucose (< 40 mg/dl or > 125 mg/dl).

All neonates in our hospital were observed in the obstetrics-neonatal ward for a minimum of 2 days following birth. The presence of relevant clinical features was determined through monitoring by the neonatology medical staff. Additionally, neonates born to GBS colonised mothers underwent two haematological examinations at 6 h and 24 h after birth. These examinations included WBC, neutrophil percentage, neutrophil count, CRP, platelet count, blood glucose monitoring, and PCT testing as required. The sepsis group, or case group, was defined as samples who had clinical manifestations of sepsis with two or more positive non-specific haematological findings.

### Statistical analysis

Categorical variables have been reported as numbers and proportions, and compared using a chi-square test or Fisher exact test. Multivariable logistic regression analysis was used to identify the optimal variables for the construction of the prediction model. These variables were expressed as odds ratios (ORs) with 95% confidence intervals (CIs) and P-values. The Bootstrap method was used for internal validation 500 times, and the receiver operating characteristic (ROC) of the model was drawn via the ggplot2 package and the pROC package, The area under the curve (AUC) and calibration curves were used to assess the performance of the prediction model. The decision curve analysis (DCA) was used to evaluate the clinical usefulness of the model. A nomogram was developed to visualize the prediction model in a user-friendly manner. To evaluate the model, data from subsequent periods of the Institute's duration in the hospital were incorporated as test data, with the modeling set and test set maintaining an approximate ratio of 8:2. Data processing and analysis were performed using R version 4.3.0 (2023-04-21). A P-value < 0.05 was considered to indicate statistical significance.

### Ethics approval and consent to participate

This study was approved by the Medical Ethics Committee of Zhejiang Provincial People’s Hospital (approval No. QT2023194) and was conducted in accordance with the Declaration of Helsinki. The study was retrospective and the Medical Ethics Committee of Zhejiang Provincial People’s Hospital agreed to a statement exempting patients from informed consent.

## Results

### Baseline characteristics and optimal risk factors identification

The baseline characteristics of the sample are summarized in Table 2. A total of 339 mother-infant pairs, including 84 patients and 255 controls, were included in this study. GBS was detected in the rectum or vagina alone in 163 cases and both the rectum and vagina in 176 cases. All mothers were given antibiotics strictly per CDC guidelines during delivery, with 66 cases receiving antibiotics ≥ 4 times and 273 receiving antibiotics < 4 times. The two groups had no significant differences regarding gestational age, amniotic fluid index, and maternal temperature. There were five variables (maternal age, GDM, forceps-assisted delivery, umbilical cord winding, newborn gender) with statistically significant differences (P < 0.05) in the chi-square test.

**Table 2:** Baseline characteristics of the modelling cohort (n = 339).

Risk factors	Controls (n = 255)	Patients (n = 84)	Statistic	P value
Maternal characteristics				
Maternal age, years			χ^2^ = 4.561	0.033*
< 26	26 (10.2%)	16 (19.1%)		
≥ 26	229 (89.8%)	68 (80.9%)		
GBS vagina ( +) and perianal ( +)	130 (50.98%)	46 (54.76%)	χ^2^ = 0.362	0.547
HDP	5 (1.96%)	3 (3.57%)	χ^2^ = 0.184	0.415^#^
GDM	30 (11.76%)	18 (21.43%)	χ^2^ = 4.855	0.028*
AIP	19 (7.45%)	3 (3.57%)	χ^2^ = 1.567	0.211
HIP	55 (21.57%)	17 (20.24%)	χ^2^ = 0.067	0.796
Previous history of abdominal surgery	14 (5.49%)	9 (10.71%)	χ^2^ = 2.727	0.099
Native	179 (70.20%)	57 (67.86%)	χ^2^ = 0.163	0.686
Perinatal characteristics				
Intrapartum fever (≥ 38 ℃)	12 (4.71%)	6 (7.14%)	χ^2^ = 0.340	0.388
Intrapartum antibiotic (times)			χ^2^ = 0.042	0.837
< 4	206 (80.78%)	67 (79.76%)		
≥ 4	49 (19.22%)	17 (20.24%)		
Amniotic fluid			χ^2^ = 0.403	0.525
Clear; Grade1; Grade2	231 (90.59%)	78 (92.86%)		
Grade3	24 (9.41%)	6 (7.14%)		
Oxytocin induced labor	143 (56.08%)	50 (59.52%)	χ^2^ = 0.306	0.580
PROM ≥ 18h	11 (4.31%)	3 (3.57%)	χ^2^ = 0.038	0.257^#^
AROM	120 (47.06%)	41 (48.81%)	χ^2^ = 0.078	0.780
Forceps assisted delivery	4 (1.57%)	6(7.14%)	χ^2^ = 5.049	0.017*^#^
Intrauterine distress	32 (12.55%)	13(15.48%)	χ^2^ = 0.470	0.493
Umbilical cord winding	72 (28.24%)	35(41.67%)	χ^2^ = 5.277	0.022*
Neonatal characteristics				
Gender			χ^2^ = 3.939	0.047*
Male	123 (48.24%)	51(60.71%)		
Female	132 (51.76%)	33(39.29%)		
Birth weight (g)			χ^2^ = 0.000	1.000
2500–4000	241 (94.51%)	80 (95.24%)		
< 2500 and > 4000	14 (5.49%)	4 (4.76%)		
GA (weeks)			χ^2^ = 0.787	0.375
< 40	150 (58.82%)	54 (64.19%)		
≥ 40	105 (41.18%)	30 (35.71%)		

GBS vagina ( +) and perianal( +) = GBS colonization in the maternal genital tract (vagina and perianal area); Umbilical cord winding was assessed as cord around the neck, cord around the body.

*GA* gestational age, *AROM* artificial rupture of membranes, *PROM* premature rupture of membrane, *GDM* gestational diabetes mellitus, *HDP* hypertensive disorders of pregnancy, *AIP* anaemia in pregnancy, *HIP* hypothyroidism in pregnancy.

*Significant at P < 0.05 level, ^#^The counting variable has a theoretical number < 10, implies the use of Fisher's exact probability and the remainder using the chi-square test.

### Univariate regression and multivariate equation modeling

The results of the univariate analysis are shown in Table 3. It showed that independent factors affecting neonatal sepsis included maternal age < 26 years, maternal gestational diabetes, whether forceps assisted delivery, whether umbilical cord winding, and neonatal gender.

**Table 3:** Univariate logistic regression models in the group.

Variable	Variable contrast	OR (95% CI)	P value
Maternal age, years	≥ 26	Reference	0.034*
	< 26	2.16 (1.06–4.09)	
GBS vagina ( +) and perianal ( +)	No	Reference	0.548
	Yes	1.16 (0.71–1.91)	
HDP	No	Reference	0.406
	Yes	1.85 (0.43–7.92)	
GDM	No	Reference	0.030*
	Yes	2.17 (1.07–3.90)	
AIP	No	Reference	0.221
	Yes	0.46 (0.13–1.60)	
HIP	No	Reference	0.796
	Yes	0.92 (0.50–1.70)	
Previous history of abdominal surgery	No	Reference	0.105
	Yes	2.07 (0.86–4.96)	
Native	No	Reference	0.686
	Yes	0.90(0.53–1.52)	
Intrapartum fever	< 38 ℃	Reference	0.391
	≥ 38 ℃	1.56 (0.57–4.29)	
Intrapartum antibiotic, times	< 4	Reference	0.837
	≥ 4	0.94 (0.51–1.74)	
Amniotic fluid	Clear; Grade1; Grade2	Reference	0.527
	Grade3	0.74 (0.29–1.88)	
Oxytocin induced labor	No	Reference	0.580
	Yes	1.15 (0.70–1.90)	
PROM ≥ 18 h	No	Reference	0.767
	Yes	0.82 (0.22–3.02)	
AROM	No	0.93 (0.57–1.53)	0.781
	Yes	Reference	
Forceps assisted delivery	No	Reference	0.032*
	Yes	3.76 (1.72–5.21)	
Intrauterine distress	No	0.78 (0.39–1.57)	0.494
	Yes	Reference	
Umbilical cord winding	No	Reference	0.023*
	Yes	1.75 (1.32–2.67)	
Newborn gender	Female	Reference	0.048*
	Male	1.66 (1.01–2.74)	
Birth weight	2500–4000 g	Reference	0.796
	< 2500 g and > 4000 g	0.86 (0.28–2.69)	
GA, weeks	≥ 40	Reference	0.376
	< 40	1.26 (0.76—2.10)	

GBS vagina( +) and perianal( +) = GBS colonization in the maternal genital tract (vagina and perianal area); Umbilical cord winding was assessed as cord around the neck, cord around the body.

*CI* confidence interval, *GA* gestational age, *AROM* artificial rupture of membranes, *PROM* premature rupture of membrane, *GDM* gestational diabetes mellitus, *HDP* hypertensive disorders of pregnancy, *AIP* anaemia in pregnancy, *HIP* hypothyroidism in pregnancy.

*Significant at P < 0.05 level.

The results of the study showed that for mothers with definite GBS colonization.

**Figure 4:**
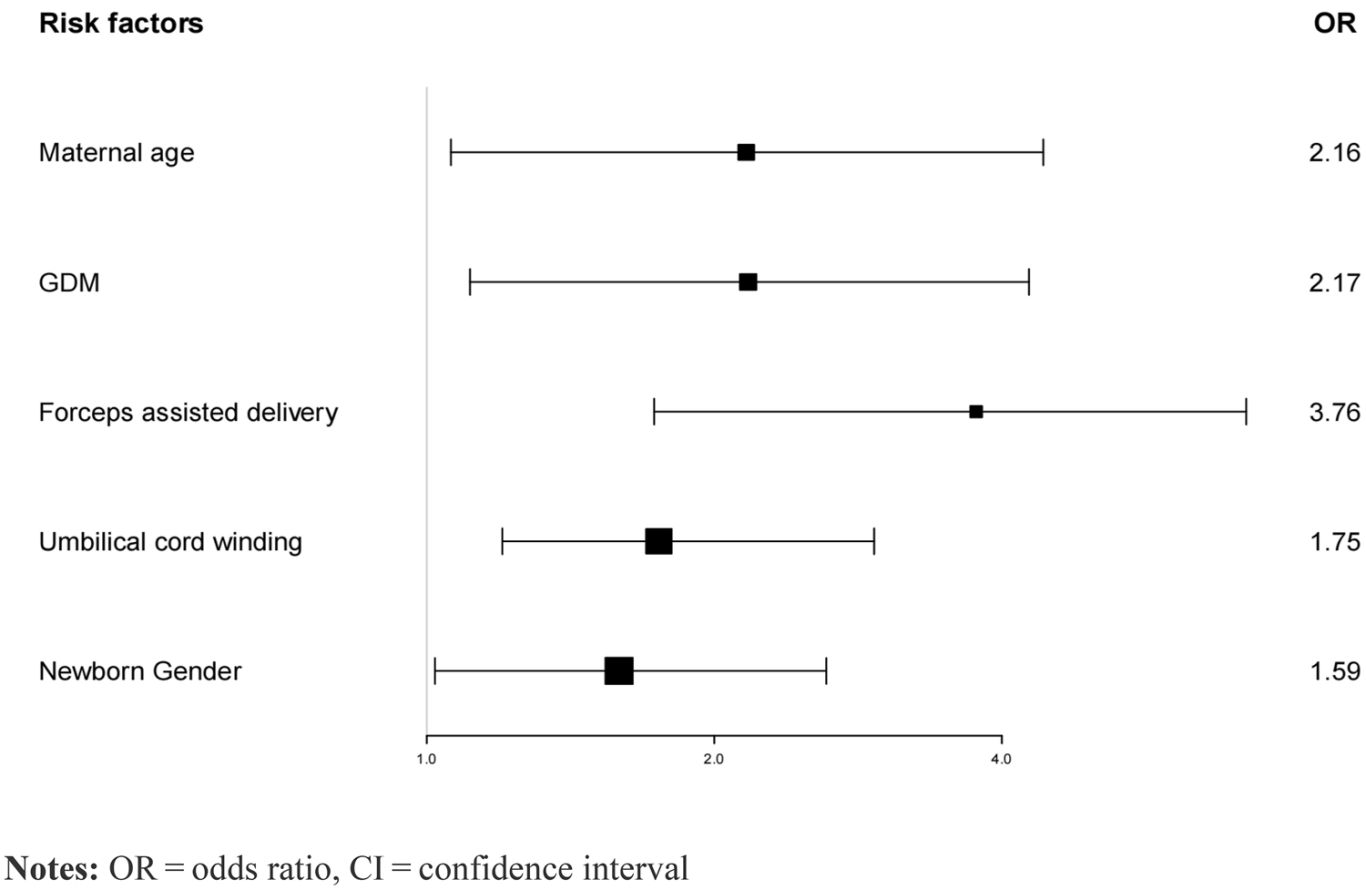
The risk factors in multivariable logistic regression analysis.

Baseline variables that were with univariate relationship with outcome in Table 3 were entered into multivariate regression model. Variables for inclusion were carefully chosen, given the number of events available, to ensure parsimony of the final model. Further determined the corresponding odds ratios (OR).﻿ The findings indicate a statistically significant elevation in the corrected odds ratio (OR) of neonatal infections among newborns born to women who were younger than 26 years old, with an approximate increase of 2.16 times (OR = 2.16, 95% CI 1.06–4.42, p = 0.034). Furthermore, there was a significant rise of approximately 2.17 times in the corrected odds ratio (OR) of infections in newborns born to women with gestational diabetes compared to those born to women who had normal blood glucose levels during pregnancy (OR = 2.17, 95% CI 1.11–4.27, p = 0.024). The adjusted odds ratio (OR) for infections was found to be significantly higher in newborns delivered with the use of forceps compared to those delivered without forceps-assisted reproduction, with a factor of approximately 3.76, (OR = 3.76, 95% CI 1.72–5.21, p = 0.032). Additionally, the adjusted odds ratio (OR) for infections in neonates with umbilical cord entanglement during labor was significantly higher by approximately 1.75 times (OR = 1.75, 95% CI 1.32–2.67, p = 0.041) compared to neonates without umbilical cord winding. Lastly, the adjusted odds ratio (OR) for neonatal infections in male neonates was found to be significantly higher, with a magnitude of approximately 1.59 times (OR = 1.59, 95% CI 1.00–2.62, p = 0.050). The results of the multivariable logistic regression analysis are displayed as forest plots in Fig. 4.

### Establishment of nomogram and prediction model evaluation

**Figure 5:**
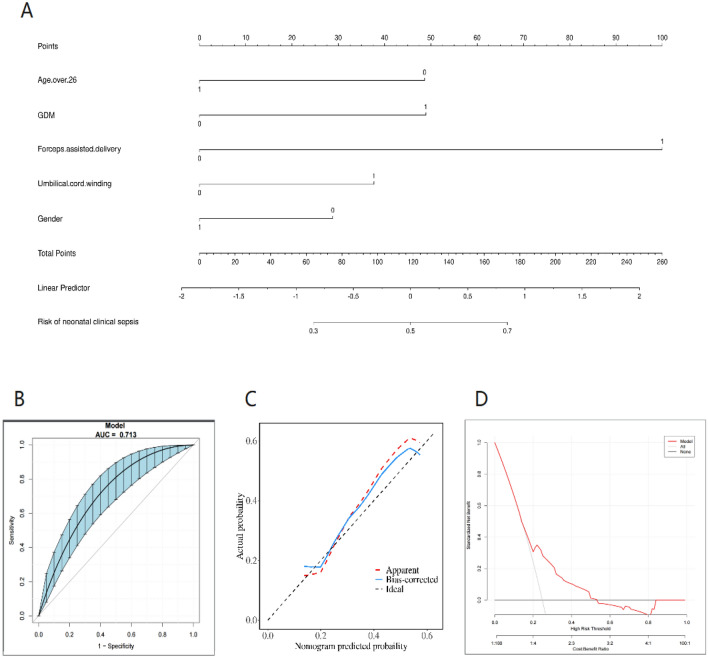
(**A**) Nomogram for the prediction of neonatal sepsis. The nomogram consists of graph lines that include risk factors (maternal age, GDM,forceps assisted delivery,umbilical cord winding,newborn gender), individual scores (Points), total scores (Total Points), and event risk (neonatal clinical sepsis). The line segment corresponding to each risk factor is marked with a scale, which represents the range of possible values of the factor, and the length of the line segment reflects the contribution of the factor to the outcome event. “Points” at the top of the graph indicate the corresponding scores of risk factors under different values. The total score of all the individual scores of the risk factors is “Total Points”, which corresponds to “neonatal clinical sepsis” at the bottom of the graph, which represents the predicted probability of progression to neonatal clinical sepsis during hospitalisation. GDM gestational diabetes mellitus. (B) ROC curve for modelling set (Bootstrap resampling times = 500). The area under the ROC curve (AUC) was 0.713 (95% CI 0.635–0.773). Blue shading shows the bootstrap estimated 95% CI with the AUC. *ROC* receiver operating characteristic, *AUC* area under the ROC. (**C**) Calibration curve of the nomogram. The x-axis represents the risk predicted by the nomogram. The y-axis represents the patients diagnosed with neonatal sepsis. The diagonal dotted line represents a perfect prediction by an ideal model. The apparent line represents the performance of the nomogram. (**D**) Decision curve analysis in prediction of neonatal sepsis. The black line represents no neonatal sepsis in all infants and a net profit of 0;The gray line represents the occurrence of neonatal sepsis in all infants, and the net gain rate is the slope of the inverse sloping line.The red line represents the DCA curve of this model. When the threshold probability of high risk is between the gray line and the red line (0.25–1.0), it is feasible for this model to predict the occurrence of neonatal sepsis, and the net gain rate of children is high.

The five independent risk factors identified through logistic multivariate regression analysis were used to develop an individualized nomogram model for the prediction of neonatal sepsis (Fig. 5A). For instance, when considering various factors such as the maternal age below 26 years (50 points), absence of gestational diabetes during pregnancy (0 points), forceps delivery (100 points), umbilical cord entanglement at delivery (38 points), and male neonatal gender (32 points), the cumulative score of 220 points is indicative of the neonatal clinical sepsis risk axis. Consequently, the estimated risk of neonatal clinical sepsis for this particular child is approximately 80%. We plotted the ROC curve of the predicted probability and calculated its AUC value. The AUC value of the model for distinguishing the risk of neonatal clinical sepsis was 0.713 (95% CI 0.635–0.773), indicating that the model has good predictive ability (Fig. 5B)^16^. At the same time, The calibration curve illustrated an overlap between the probabilities of the predicted and actual diagnosis of neonatal sepsis (Fig. 5C), which revealed that the observed and expected values showed good consistency. Finally, we plotted the DCA curve of the model using high-risk threshold probability as the x-axis and net profit rate as the y-axis (Fig. 5D), indicating that using this nomogram model to evaluate the high-risk of clinical sepsis in newborns with GBS-positive mothers yielded a net clinical benefit. Besides, the DCA curve showed that the model has good clinical utility. The optimal metric threshold for the classification model is determined through calculation, with a maximum index of 0.305 serving as the optimal indicator threshold. Upon reaching this diagnostic cut-off value, the model exhibits a sensitivity size of 0.686 and a specificity size of 0.619, resulting in the most effective evaluation. In instances where the risk of neonatal sepsis exceeds 30.5% as calculated by the risk calculator, additional monitoring and attention are necessary, Given the unique and expeditious development of neonatal physiology, we have reached a consensus that establishing a risk threshold model of 30.5% aligns with the clinical diagnostic and treatment features.

### Timing verification

**Figure 6:**
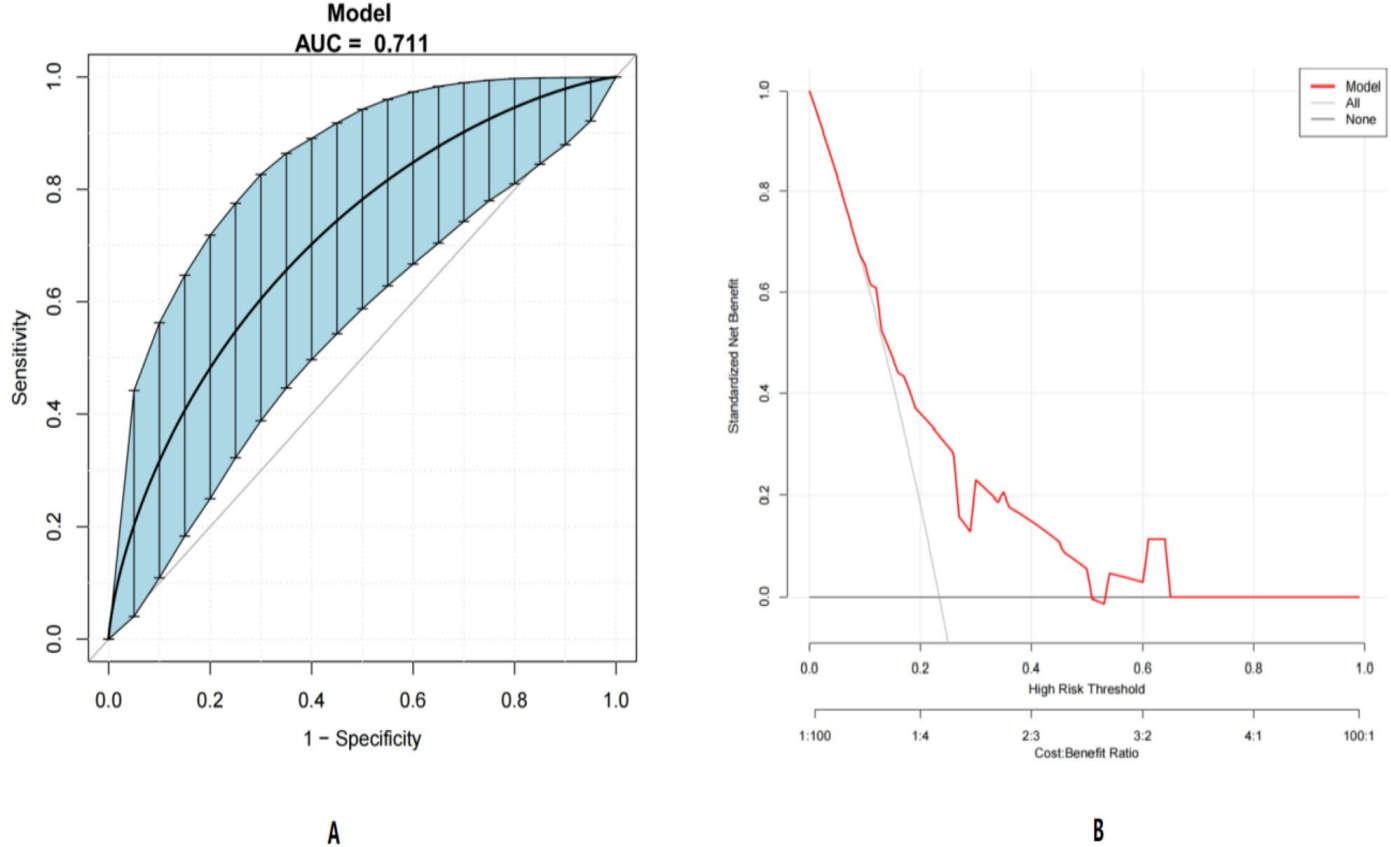
Validation set related charts. (**A**) ROC curves for the validation set (Bootstrap resampling times = 500). The area under the ROC curve (AUC) was 0.711, (95% CI 0.592–0.808). Blue shading shows the bootstrap estimated 95% CI with the AUC. (**B**) DCA curves for the validation set. The black line represents no neonatal sepsis in all infants and a net profit of 0;The gray line represents the occurrence of neonatal sepsis in all infants, and the net gain rate is the slope of the inverse sloping line.

A summary of the datasets used for training and testing is shown in Table 4 and Fig. 6.We only used the test set as a performance evaluation. By studying 77 mother–child pairs of GBS colonised mothers and their born neonates eligible for enrolment in the hospital 2023.4.1–2024.1.1 were included as test data, the sample ratio between the modelling set and the test set was roughly in the ratio of 8:2. In the internal validation cohort, the AUC value of the model was 0.711, 95% CI (0.592–0.808).

**Table 4:** Modelling sets and validation sets.

	Sample size	AUC	95% CI
Modelling sets	N = 339	0.713	0.635–0.773
Validation sets	N = 77	0.711	0.592–0.808

## Discussion

### Main findings

In this study, we established a hitherto undocumented predictive model for the probability of neonatal sepsis in infants born to GBS-colonized mothers for early prediction and intervention of newborn disease status by neonatologists during the 2–3 day hospital observation period after vaginal delivery. In the accompanying supplementary materials, we provide a web calculator that utilizes the pertinent factors of the nomogram to compute neonatal sepsis. This calculator, which we have developed and designed, facilitates the comparison of the probability of neonatal sepsis and cut-off value, thereby enabling the early identification of neonates at heightened risk of sepsis. This, in turn, empowers healthcare professionals to conduct further laboratory tests and administer appropriate antibacterial interventions.

Our study reported maternal age < 26, maternal gestational diabetes mellitus, forceps-assisted delivery, umbilical cord wrapped around the neck or body, and neonatal sex of male as risk factors affecting the outcome of neonatal sepsis. Here is some of our discussion on risk factors:

### Maternal age

In previous related studies, the general belief was that the older the mother's age, the more adverse effects it would have on the newborn. However, our study on clinical sepsis in neonates born to GBS-colonized mothers found that the younger the maternal age, the higher the probability of clinical sepsis in neonates. This discrepancy may be related to heterogeneity in maternal age at delivery, education levels of women in China, age of first childbirth for women in urban and rural areas, inadequate prenatal care and health education plans due to a younger age at first childbirth, and economic status, among other factors. A cross-sectional population study in rural northwestern China found that infection during pregnancy was associated with an increased risk of poor birth outcomes among young, less educated, and poor pregnant women^17^. Across all cohorts from 12 European countries, babies from less educated mothers were born in poor health^18^. Younger mothers may not have enough experience or knowledge about pregnancy and may be less aware of potential risks or danger signals during pregnancy. Therefore, we suggest that greater attention should be paid to younger GBS-colonized mothers, and more comprehensive GBS-related health education should be provided during antenatal checkups.

### GDM

Our findings suggest that newborns of GDM-diagnosed mothers with GBS colonization are more likely to develop sepsis. Maternal gestational diabetes mellitus has been shown to lead to increased lower genital tract infectiousness and increased rates of neonatal sepsis infection^19^. Mitanchez et al.'s study on the perinatal complications of infants born to GDM mothers showed that untreated moderate or severe GDM increased the risk of neonatal complications^20^. Kantorowska et al.'s retrospective cohort study of 176 mothers showed that improved remote patient monitoring of GDM mothers' blood glucose control could improve neonatal outcomes and reduce the incidence of neonatal sepsis^21^. A study that compared healthy pregnancies and well-controlled GDM in neonatal outcomes (NICU admission rate) showed no differences^22^. Meanwhile, our study suggests that GBS colonization in pregnant women with GDM during pregnancy increases the risk of neonatal sepsis, consistent with Mitanchez et al.'s findings.

### Forceps assisted delivery

Previous studies have shown that forceps-assisted delivery increases the likelihood of neonatal cranial injury and morbidity^23^. Given that different instruments are used in different regions for assisting natural delivery^24^, this factor may vary among different hospitals. Using forceps during fetal delivery increases contact between external instruments, the maternal reproductive tract, and the fetus. Our study showed that forceps-assisted delivery increased the probability of neonatal sepsis, considering that the study population consists of GBS-colonized mothers. Therefore, we advocate that attention must be paid to the dynamic changes of mothers and newborns during the delivery process to reduce the incidence and mortality of neonatal sepsis, and forceps-assisted vaginal delivery should be used with caution.

### Umbilical cord winding

Another predictive factor for neonatal sepsis was umbilical cord winding. Previous research has shown that fetal extracorporeal umbilical cord entanglement and torsion may obstruct umbilical blood flow and cause fetal asphyxia and adverse outcomes^25^. A retrospective analysis of factors related to fetal death during the perinatal period in 105 cases by Chitra T in 2012 showed that 86 cases (81.9%, 86/105) were related to umbilical cord/placental abnormalities^26^. The currently accepted theory is that umbilical cord torsion and entanglement lead to changes in umbilical cord plasma interleukin-6 concentration and C-reactive protein, increasing neonatal sepsis^27^. In our study, the results showed that combined umbilical cord entanglement during delivery was a risk factor for neonatal sepsis. The risk of adverse outcomes associated with cord knots, and entanglement^28^. We advocate that umbilical cord winding increases the difficulty of the fetus passing through the birth canal and increases the chance of the fetus contacting the GBS colonized birth canal during delivery.

### Gender

Infant gender was found to be another factor influencing the risk of neonatal sepsis. By reviewing the literature, we collected the results of a prospective cohort study in 2023, which showed that adverse perinatal outcomes (APO) were associated with the male gender in neonates (OR 1.92, 95% CI 1.33–2.78)^29^. A study in Korea investigated the perinatal outcomes of twins and found that males were a risk factor for neonatal neurological impairment based on logistic regression analysis in single chorionic membrane twins^30^. The above literature indicates that being male may lead to adverse perinatal and neurological outcomes. Consistently, our study showed male gender increases the probability of sepsis in neonates.

Our study constructed a nomogram through the above five variables, showed the highest discriminative ability, calibration, and clinical value in the nomogram.

### New evidence: before and after IAP

Literature reports suggest intrapartum fever (≥ 38 ℃), delivery before 37 weeks of gestation, membrane rupture ≥ 18 h, delivery of a GBS-infected infant, and GBS bacteriuria during pregnancy are risk factors for neonatal sepsis. Longitudinal comparisons with the era before universal screening for GBS and intrapartum antibiotic prophylaxis (IAP) (before 2005) have helped to identify these factors^31–34^. However, a study comparing two methods for identifying candidates for prophylactic antibiotic treatment during delivery found that nearly 50% of women with early-onset GBS disease in their newborns did not have any of the aforementioned risk factors^35^. More recently, a study from India showed that the factors influencing GBS sepsis changed significantly before and after IAP implementation; premature birth, premature rupture of membranes, and rupture of membranes for ≥ 18 or 24 h were significant risk factors before 2003 but had no significant effect after IAP implementation^36^. The above studies suggest that antibiotics have significantly altered the risk factors for neonatal sepsis, which is consistent with our research results. Similarly, our study revealed that membrane rupture and its duration are no longer significant risk factors for neonatal sepsis. It is now understood that premature rupture of membranes (PROM) is related to vaginal dysbiosis, and continuous upward colonization, infection, and inflammation can lead to the occurrence of early-onset neonatal sepsis (EONS)^37^. With the full application of IAP, membrane rupture no longer significantly impacts the microbial environment of the reproductive tract, thereby reducing the incidence of neonatal sepsis. Given that the study population consists of special mothers colonized with GBS, previous studies have shown that the enzymes encoded by GBS genes are NADH peroxidases, which can defend against reactive oxygen species stress derived from macrophages and promote uterine infection during pregnancy, leading to the occurrence of neonatal sepsis^38^. The prophylactic use of antibiotics reduces the occurrence of uterine infection and neonatal sepsis. At the same time, intrapartum fever is no longer a significant risk factor for neonatal sepsis in this study. Policies such as active temperature measurement and prophylactic antibiotic use during delivery for women admitted to the hospital for delivery have reduced the incidence of maternal fever. The average 3–5 days of waiting time and close monitoring of the obstetric medical team during waiting time have also reduced the impact of maternal fever on neonatal outcomes. During the initial processing stage of the study population, we excluded cases of premature birth and emergency delivery to avoid the influence of other complicated delivery situations. We only evaluated clinical data on whether late preterm and term infants developed sepsis and found no relationship between gestational age and neonatal sepsis.

### Model construction and evaluation

In this study, we evaluated the perinatal predictive factors for newborn sepsis in GBS-colonized mothers and developed a risk prediction model for early prediction and intervention of newborn disease status by neonatologists during the 2–3 day hospital observation period after vaginal delivery. We established a visual and personalized nomogram model, providing clinical doctors with a simple and objective practical prediction tool. The model's discrimination, calibration, and clinical effectiveness were evaluated, and the AUC of the ROC curve of the model in this study was 0.713 (95% CI 0.635–0.773), indicating its ability to distinguish between newborns born to GBS-colonized mothers who develop clinical sepsis. The binary classification results were evaluated by calculating the AUC values, which indicated that the model has good discriminatory and predictive accuracy. Besides, the DCA curve showed that the model has good clinical utility.

### Driven web applications

Our findings support the previous literature regarding GDM, forceps-assisted delivery, umbilical cord winding, and newborn gender. It also provides some new evidence showing that the factors influencing neonatal sepsis changed significantly before and after IAP implementation; premature birth, premature rupture of membranes, and rupture of membranes for ≥ 18 or 24 h were significant risk factors before^32^, but had no significant effect after IAP implementation^36^. The above studies suggest that antibiotics have significantly altered the risk factors for neonatal sepsis, which is consistent with our research results. To assist neonatologists in the monitoring and management of neonates online, we have developed a web-based clinical decision support system utilizing the nomogram. The code for our proposed models can be publicly accessed at https://somehow0529.github.io/GBS-colonized_mothers_neonatal_sepsis_Prediction/. By inputting the case histories of maternal, perinatal, and neonatal correlates into the calculator on our webpage, the probability of sepsis occurring in a neonate born to a GBS-colonized mother can be determined. The web calculator and instructions for its use may also be reflected in Supplementary Material 1. There is no need to enter any information that is considered to be private, except for feature-related information, and the entered information is deleted immediately after the prediction results are generated, so there is no risk of exposing the information. This code can also be reflected in Supplementary Material 2.

### Limitations

It is imperative to underscore that the prediction model was specifically designed for the Chinese population. Despite its thorough validation, the model's performance is expected to decline due to variations in environmental disease risk factors, unmeasured risk factors, therapeutic interventions, and evolving treatment contexts across international settings. Consequently, calibration drift becomes a concern, necessitating the continuous evolution and dynamic updating of the clinical prediction models presented in this study. Therefore, prior to their application in different cultural contexts, rigorous validation is essential. Furthermore, our model is derived from a single-centre study utilizing a retrospective cohort design. Although the model's effectiveness has been assessed through time-series validation, it is important to note that this validation process was conducted internally. Therefore, it is imperative to conduct high-quality, prospective multicentre studies in the future to thoroughly examine the model's validity. Additionally, it is worth mentioning that the sample population in our study excluded individuals with gestational weeks below 36 and those with complicated delivery situations. Consequently, the generalizability of the model to the wider population is limited.

## Conclusion and outlook

In summary, our nomogram can be used to predict the likelihood of sepsis in newborns born to GBS-colonised mothers with increased accuracy, good clinical utility, and more precise prognosis prediction compared to conventional methods. Predictors that have been identified, including maternal age, GDM, umbilical cord winding, forceps-assisted delivery, and neonatal sex, possess the potential to inform clinical decision-making and antibiotic dosage adjustments in the management of neonates born to mothers colonized with GBS in the Neonatal Intensive Care Unit (NICU). Subsequent research endeavors should prioritize the validation and enhancement of this model across diverse populations, while also investigating the incorporation of additional pertinent predictors to enhance the overall performance of the model.

### Supplementary Information

Supplementary Information 1.

Supplementary Information 2.

## Data Availability

The datasets used and/or analysed during the current study available from the corresponding author on reasonable request.

## Abbreviations

EONS: Early-onset neonatal sepsis
NICU: Neonatal intensive care unit
GA: Gestational age
AROM: Artificial rupture of membranes
PROM: Premature rupture of membrane
GDM: Gestational diabetes mellitus
ICP: Intrahepatic cholestasis of pregnancy
HDP: Hypertensive disorders of pregnancy
AIP: Anaemia in pregnancy
HIP: Hypothyroidism in pregnancy
WBC: White blood cell
CRP: C-reactive protein
PCT: Procalcitonin
ROC: Receiver operating characteristic
DCA: Decision curve analysis
