# Supplementary material for: Development of risk prediction nomogram for neonatal sepsis in Group B Streptococcus-colonized mothers: a retrospective study
Source: Sci Rep. 2024 Mar 7;14:5629. doi: 10.1038/s41598-024-55783-2 (PMC10920653; doi:10.1038/s41598-024-55783-2)

**Web Calculator Description**

To assist neonatologists in the monitoring and management of neonates online, we have developed a web-based clinical decision support system utilizing the nomogram. The code for our proposed models can be publicly accessed at https://somehow0529.github.io/GBS-colonized_mothers_neonatal_sepsis_Prediction/. By inputting the case histories of maternal, perinatal, and neonatal correlates into the calculator on our webpage, the probability of sepsis occurring in a neonate born to a GBS-colonized mother can be determined.

**Instructions for use**

This is a web calculator for calculating the risk of sepsis in newborns born to mothers with GBS implants. The five influencing factors listed above are filtered by logistic regression. The pediatrician can automatically calculate the probability of sepsis in the newborn using the spaces following the appropriate risk factors based on the specific clinical conditions of the mother and the newborn.

**Special note**

In the gender column, FEMALE=1, and MALE=0; select 0 or 1; for the other corresponding factors, where the relevant factor appears=Yes and the relevant factor does not appear=No;

**Legends and representations**

For example, if the mother used forceps during labor, forceps-assisted delivery = Yes; if the mother was 28 years old, age over 26 = Yes; if the newborn's sex was female, GENDER = 1; if the newborn was born without umbilical cord wrapping around the neck, UMBILICAL CORD WINDING= No; if the mother had gestational diabetes, then GDM= Yes; shows a probability of 61.60% for the corresponding neonatal sepsis.


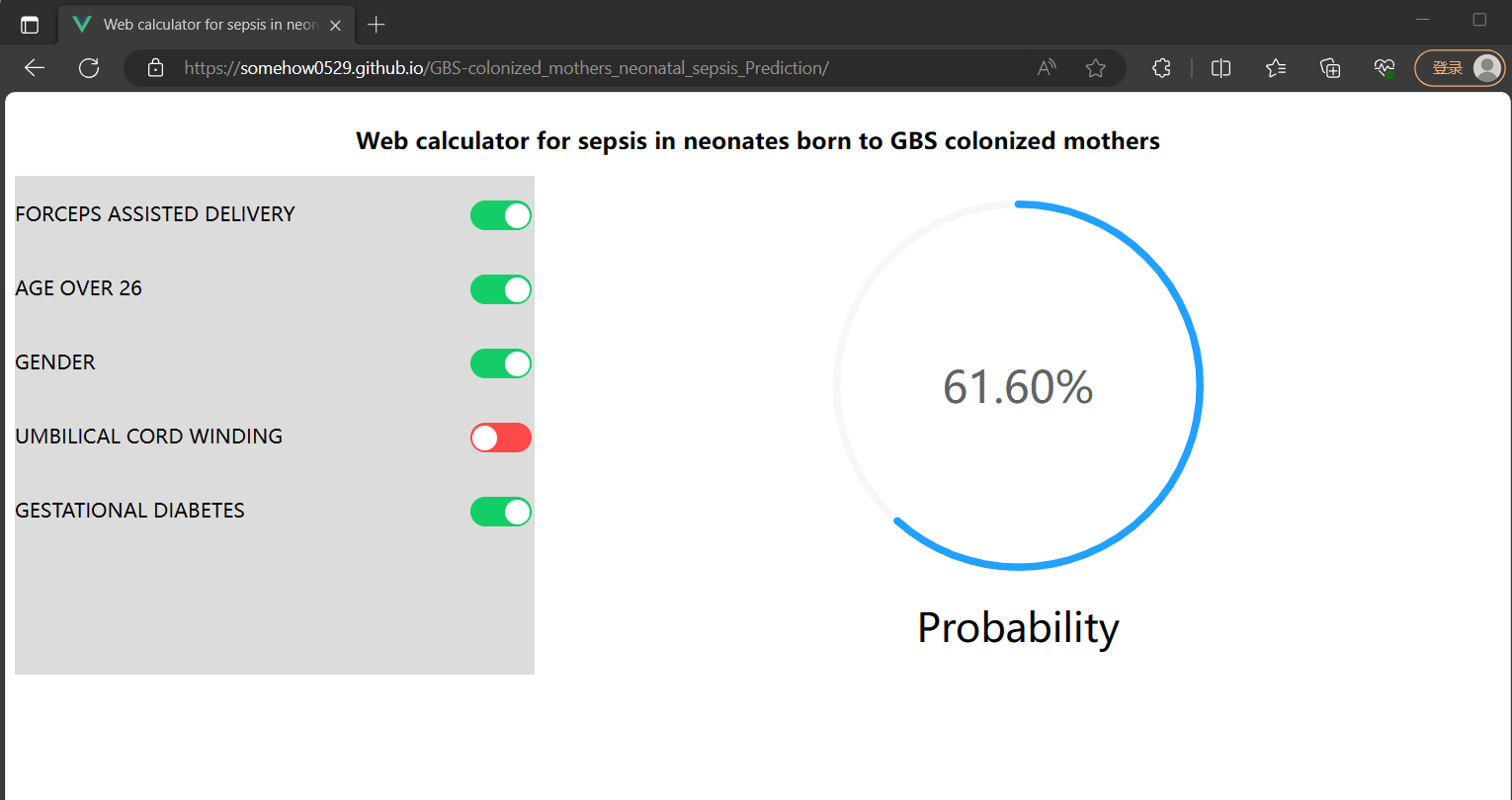

Supplement: Supplementary file 1 — Supplementary Information 1. [file 41598_2024_55783_MOESM1_ESM.docx]
