# Supplementary material for: Development of risk prediction nomogram for neonatal sepsis in Group B Streptococcus-colonized mothers: a retrospective study
Source: Sci Rep. 2024 Mar 7;14:5629. doi: 10.1038/s41598-024-55783-2 (PMC10920653; doi:10.1038/s41598-024-55783-2)
Supplement: Supplementary file 2 — Supplementary Information 2. [file 41598_2024_55783_MOESM2_ESM.zip › GBS-colonized_mothers_neonatal_sepsis_Prediction-master/index.html]

Web calculator for sepsis in neonates born to GBS colonized mothers **We're sorry but mq\_project doesn't work properly without JavaScript enabled. Please enable it to continue.**
